# Prevalence and Effect of Cerebral Small Vessel Disease in Stroke Patients With Aspirin Treatment Failure–A Hospital-Based Stroke Secondary Prevention Registry

**DOI:** 10.3389/fneur.2021.645444

**Published:** 2021-04-13

**Authors:** Ping-Song Chou, Pi-Shan Sung, Chi-Hung Liu, Yueh-Feng Sung, Ray-Chang Tzeng, Chun-Pai Yang, Chi-Hsun Lien, Helen L. Po, Shang-Chang Ho, Yi-Te Tsai, Tsang-Shan Chen, Shey-Lin Wu, Han-Hwa Hu, A-Ching Chao

**Affiliations:** ^1^Department of Neurology, Kaohsiung Medical University Hospital, Kaohsiung Medical University, Kaohsiung, Taiwan; ^2^Department of Neurology, Faculty of Medicine, College of Medicine, Kaohsiung Medical University, Kaohsiung, Taiwan; ^3^Department of Neurology, National Cheng Kung University Hospital, College of Medicine, National Cheng Kung University, Tainan, Taiwan; ^4^Department of Neurology, Stroke Center, Linkou Chang Gung Memorial Hospital, Taoyuan, Taiwan; ^5^College of Medicine, Chang Gung University, Taoyuan, Taiwan; ^6^Department of Neurology, National Defense Medical Center, Tri-Service General Hospital, Taipei, Taiwan; ^7^Department of Neurology, Tainan Municipal Hospital (Managed by Show Chwan Medical Care Corporation), Tainan, Taiwan; ^8^Department of Neurology, Kuang Tien General Hospital, Taichung, Taiwan; ^9^Department of Nutrition, Huang-Kuang University, Taichung, Taiwan; ^10^Department of Neurology, Tungs' Taichung Metro Harbor Hospital, Taichung, Taiwan; ^11^Department of Neurology, Stroke Center, MacKay Memorial Hospital, Taipei, Taiwan; ^12^Department of Neurology, National Taiwan University Hospital Yunlin Branch, Yunlin, Taiwan; ^13^Department of Neurology, Sin-Lau Hospital, The Presbyterian Church of Taiwan, Tainan, Taiwan; ^14^Department of Neurology of Changhua Christian Hospital, Changhua, Taiwan; ^15^Department of Neurology, Taipei Medical University-Shaung Ho Hospital, Taipei, Taiwan; ^16^Beijing Tiantan Hospital, Capital Medical University, Beijing, China; ^17^Advanced Innovation Center for Human Brain Protection, Capital Medical University, Beijing, China

**Keywords:** aspirin treatment failure, cerebral small vessel disease, microbleed, stroke, white matter lesion

## Abstract

**Background:** Breakthrough strokes during treatment with aspirin, termed clinical aspirin treatment failure (ATF), is common in clinical practice. The burden of cerebral small vessel disease (SVD) is associated with an increased recurrent ischemic stroke risk. However, the association between SVD and ATF remains unclear. This study investigated the prevalence and clinical characteristics of SVD in stroke patients with ATF.

**Methods:** Data from a prospective, and multicenter stroke with ATF registry established in 2018 in Taiwan were used, and 300 patients who developed ischemic stroke concurrent with regular use of aspirin were enrolled. White matter lesions (WMLs) and cerebral microbleeds (CMBs) were identified using the Fazekas scale and Microbleed Anatomical Rating Scale, respectively. Demographic data, cardiovascular comorbidities, and index stroke characteristics of patients with different WML and CMB severities were compared. Logistic regression analyses were performed to explore the factors independently associated with outcomes after ATF.

**Results:** The mean patient age was 69.5 ± 11.8 years, and 70.0% of patients were men. Among all patients, periventricular WML (PVWML), deep WML (DWML), and CMB prevalence was 93.3, 90.0, and 52.5%, respectively. Furthermore, 46.0% of the index strokes were small vessel occlusions. Severe PVWMLs and DWMLs were significantly associated with high CMB burdens. Patients with moderate-to-severe PVWMLs and DWMLs were significantly older and had higher cardiovascular comorbidity prevalence than did patients with no or mild WMLs. Moreover, patients with favorable outcomes exhibited significantly low prevalence of severe PVWMLs (*p* = 0.001) and DWMLs (*p* = 0.001). After logistic regression was applied, severe WMLs predicted less favorable outcomes independently, compared with those with no to moderate PVWMLs and DWMLs [odds ratio (OR), 0.47; 95% confidence interval (CI), 0.25–0.87 for severe PVWMLs; OR, 0.40; 95% CI, 0.21–0.79 for severe DWMLs].

**Conclusions:** SVD is common in stroke patients with ATF. PVWMLs and DWMLs are independently associated with functional outcomes in stroke patients with ATF. The burden of SVD should be considered in future antiplatelet strategies for stroke patients after ATF.

## Introduction

Stroke is the second leading cause of death worldwide and results in disability in a large proportion of survivors (1). Ischemic strokes account for ~80% of strokes (2). Cerebral small vessel disease (SVD) refers to a group of pathological processes involving the small arteries, arterioles, venules, and capillaries of the brain, such as lacunar infarcts, white matter lesions (WMLs), and cerebral microbleeds (CMBs) (3). SVD plays a critical role in stroke. The total burden of SVD was reported to be positively associated with the risk of recurrent ischemic strokes and intracerebral hemorrhage (ICH) in patients with a prior transient ischemic attack (TIA) or ischemic stroke (4).

Antiplatelet therapy is recommended for non-cardioembolic ischemic stroke treatment and prevention (5). Aspirin is the most commonly used antiplatelet agent for the secondary prevention of ischemic stroke because of its low cost and availability (6). Aspirin was determined to reduce the risk of ischemic stroke by 22% in secondary prevention (7). However, patients who develop an ischemic stroke during aspirin treatment, termed clinical aspirin treatment failure (ATF), represent 30–40% and 40–50% of patients with stroke in clinical trials (8–10), and in clinical practice (11–13), respectively.

ATF has been reported to be associated with old age, high prevalence of comorbidities, history of cardiovascular disease, and prior symptomatic cerebrovascular disease (10, 13). However, the characteristics and effect of SVD in ATF remain unclear. SVD is associated with an increased risk of recurrent ischemic stroke (4). In addition, SVD is associated with cerebral endothelium dysfunction (14) and impairment of brain network connectivity (15). Therefore, we hypothesized that SVD is associated with, and could affect the functional outcome of stroke with ATF. In this research, the prevalence and clinical characteristics of WMLs and CMBs were investigated in stroke patients with ATF.

## Materials and Methods

### Participants

This study was conducted using data from a prospective, and multicenter registry of specific stroke patients with ATF, established in 2018 in Taiwan. Eligible patients were older than 20 years, developed an ischemic stroke (the index stroke) within 60 days before recruitment, and had a regular use of aspirin 7 days before the index stroke. The indication of aspirin was primary or secondary prevention of cardiovascular or cerebrovascular events, and the dose of aspirin was 50 to 100 mg per day according to the prescription of the physician. Patients who received intravenous thrombolysis and endovascular thrombectomy for the index stroke could be included. Exclusion criteria were a history of antiplatelet agents use other than aspirin, a known or high risk of cardioembolism, a history of anticoagulant use, an impaired medical adherence and contraindication to magnetic resonance imaging (MRI) examination.

A comprehensive evaluation, including demographic and medical history data collection, physical and neurological examination, blood biochemistry examination, carotid artery ultrasonography, transcranial color Doppler, and cerebral MRI with magnetic resonance angiography, was performed for each patient. The hypertension referred to either a diastolic and systolic blood pressure of ≥90 and ≥140 mmHg, respectively, or the use of antihypertensive medications. The hyperlipidemia referred to a total cholesterol ≥160 mg/dl or a low-density lipoprotein ≥100 mg/dl. The Essen Stroke Risk Score (ESRS), a 10-point scale derived from Clopidogrel vs. Aspirin in Patients at Risk of Ischemic Events (CAPRIE) trial (16), was applied to predict the risk of recurrent ischemic stroke after the index stroke, based on the cardiovascular comorbidities (17, 18). The diagnosis of ischemic stroke was confirmed by a neurologist based on the clinical symptoms of stroke and a cerebral MRI. Stroke severity was evaluated using the National Institutes of Health Stroke Scale (NIHSS) score, and functional outcomes were determined using the modified Rankin scale (mRS). Neurological deterioration was defined based on the increase in NIHSS scores during hospitalization or at discharge, compared with the initial NHISS. A favorable functional outcome was defined as an mRS score of 0–2 at discharge. The stroke subtype was classified according to the TOAST criteria (19).

### WML and CMB Grading

All MRIs were performed following a standardized MRI protocol, including axial diffusion-weighted imaging, axial T2-weighted fluid-attenuated inversion recovery imaging (FLAIR), and gradient recalled echo (GRE) T2^*^-weighted imaging or susceptibility-weighted imaging (SWI) with whole-brain coverage. Four experienced stroke neurologists, blinded to the clinical course and outcome of the patients, assessed the WMLs and CMBs of the cerebral MRI by using FLAIR and GRE or SWI, respectively.

WMLs were visually determined to be periventricular WMLs (PVWMLs) and deep WMLs (DWMLs) using the modified Fazekas scale (20, 21), which is the most frequently used assessment tool for WMLs. The Fazekas scale is a 4-point scale: 0 (no WML), 1 (mild WML), 2 (moderate WML), and 3 (severe WML). The definition, location, and number of CMBs were determined using a validated visual rating scale, the Microbleed Anatomical Rating Scale (MARS) (22). Patients were divided into two subgroups: with and without CMBs.

### Ethical Statements

All centers were required to receive approval from their Institutional Review Board before initiation of this study. All patients, or their legal representatives, provided written informed consent before inclusion.

### Statistical Analysis

Statistical analysis was performed using SPSS (version 22.0). All statistical tests were two-tailed, and an α ≥ 0.05 was considered significant. Analysis of variance or independent student *t*-tests were used for continuous variables and chi-squared test was used for categorical variables to evaluate the differences and trends in demographic data, cardiovascular comorbidities, and severities and subtypes of the index stroke based on WML severity and CMB presence and burden.

Independent student *t* and the chi-square tests were performed to assess the differences in WMLs and CMBs between patients with and without a neurological deterioration and favorable outcome. Furthermore, a multivariable logistic regression analysis was conducted, in which a neurological deterioration and favorable outcome were used as dependent variables to compare the effect of WMLs and CMBs on the stroke outcome after ATF, after adjustment for the effects of age, sex, ESRS scores, and TOAST classification of the index stroke.

## Results

### Study Participants

The cohort comprised 300 patients who developed an acute ischemic stroke during aspirin treatment, confirmed based on clinical symptoms and cerebral MRI. The mean age of the patients was 69.5 ± 11.8 years, and 70.0% of the patients were men. The mean ESRS score of the patients was 5.1 ± 1.3. For the index stroke, the mean initial and discharge NIHSS scores of the patients was 5.5 ± 5.0 and 4.8 ± 4.9, respectively. In the TOAST classification, 32.0% of strokes were large artery atherosclerosis and 46.0% were small vessel occlusions. The demographic characteristics, prevalence of vascular risk factors, severities of the index stroke, and presence of SVD are displayed in Table 1.

**Table 1:** Demographic characteristics of the participants.

***N*** **=** **300 (CMB** **=** **236)**	
Age, year, mean (±SD)	69.5 (±11.8)
Sex, male, *n* (%)	210 (70.0)
BMI, mean (±SD)	25.2 (±4.0)
**Medical history**	
Hypertension, yes, *n* (%)	262 (87.3)
Diabetes, yes, *n* (%)	156 (52.0)
Hyperlipidemia, yes, *n* (%)	198 (66.0)
Stroke/TIA, yes, *n* (%)	212 (70.7)
Coronary artery disease, yes, *n* (%)	49 (16.3)
Essen stroke risk score, mean (±SD)	5.1 (±1.3)
**Index stroke**	
Initial NIHSS, mean (±SD)	5.5 (±5.0)
Discharge NIHSS, mean (±SD)	4.8 (±4.9)
Neurological deterioration, yes, *n* (%)	27 (9.0)
Favorable outcome (mRS 0–2), yes, *n* (%)	158 (52.7)
**TOAST classification**	
Large artery atherosclerosis, *n* (%)	96 (32.0)
Small vessel occlusion, *n* (%)	138 (46.0)
**Fazekas Scale (periventricular)**, ***n*** **(%)**	
0	20 (6.7)
1	74 (24.7)
2	144 (48.0)
3	62 (20.7)
**Fazekas Scale (deep white matter)**, ***n*** **(%)**	
0	30 (10.0)
1	126 (42.0)
2	90 (30.0)
3	54 (18.0)
Presence of CMB, yes, *n* (%)	124 (52.5)
Number of CMB, mean (±SD)	3.8 (±9.2)

*CMB, cerebral microbleeds; mRS, modified Rankin scale; NIHSS, National Institutes of Health Stroke Scale; SD, standard deviation; TIA, transient ischemic attack*.

### WMLs

Among all patients, 93.3% had PVWMLs, and 90.0% had DWMLs. Furthermore, 74 (24.7%), 144 (48.0%), and 62 (20.7%) patients displayed mild, moderate, and severe PVWMLs, respectively; 126 (42.0%), 90 (30.0%), and 54 (18.0%) patients had mild, moderate, and severe DWMLs, respectively. The relationships between severities of WMLs, vascular risk factors, and stroke index are presented in Tables 2, 3. The age differed significantly between patients with different severities of PVWMLs (*p*_*trend*_ < 0.001) and DWMLs (*p*_*trend*_ < 0.001). PVWML and DWML severities increased along with age. Furthermore, PVWMLs and DWMLs were significantly associated with numbers of CMBs (*p*_*trend*_ = 0.004 for PVWMLs, and *p*_*trend*_ < 0.001 for DWMLs). Patients with severe PVWMLs and DWMLs had significantly high CMB burdens. The ESRS scores varied significantly between PVWMLs and DWMLs severity groups (*p*_*trend*_ < 0.001 for both). Patients with no or mild PVWMLs and DWMLs had significantly low ESRS scores. Patients with severe PVWMLs and DWMLs showed a trend toward a high prevalence of hypertension (*p*_*trend*_ = 0.005 for PVWMLs and *p*_*trend*_ = 0.110 for DWMLs). Patients with severe PVWMLs and DWMLs had a trend toward an increase in initial and discharge NIHSS scores. Patients with moderate-to-severe PVWMLs had significantly high rates of stroke caused by small vessel occlusion, whereas the distribution of TOAST stroke subtype did not differ significantly based on DWML severity.

**Table 2:** Relationship between periventricular white matter lesions and characteristics of stroke patients with aspirin treatment failure.

**Periventricular WML**	**Absent**	**Mild**	**Moderate**	**Severe**	***p*-value**	***p* for trend**
*N*	20 (6.7%)	74 (24.7%)	144 (48.0%)	62 (20.7%)		
Age, year, mean (±SD)^a^	56.9 (±14.9)	63.9 (±10.6)	72.1 (±10.5)	74.4 (±9.8)	<0.001^**^	<0.001^**^
Sex, male, *n* (%)^b^	16 (80.0)	52 (70.3)	102 (70.8)	40 (64.5)	0.595	0.250
CMB, mean (±SD)^a^	1.9 (±7.5)	1.9 (±5.1)	2.9 (±6.9)	8.7 (±14.5)	<0.001^**^	0.004^*^
**Medical history^b^**						
Hypertension, yes, *n* (%)	11 (55.0)	66 (89.2)	129 (89.6)	56 (90.3)	<0.001^**^	0.005^*^
Diabetes, yes, *n* (%)	10 (50.0)	43 (58.1)	67 (46.5)	36 (58.1)	0.286	0.996
Hyperlipidemia, yes, *n* (%)	15 (75.0)	52 (70.3)	95 (66.0)	36 (58.1)	0.382	0.087
Stroke/TIA, yes, *n* (%)	13 (65.0)	48 (64.9)	102 (70.8)	49 (79.0)	0.307	0.073
Coronary artery disease, yes, *n* (%)	4 (20.0)	8 (10.8)	29 (20.1)	8 (12.9)	0.272	0.926
Essen stroke risk score, mean (±SD)^a^	4.1 (±1.4)	4.7 (±1.3)	5.3 (±1.2)	5.6 (±1.3)	<0.001^**^	<0.001^**^
**Index stroke^a^**						
Initial NIHSS, mean (±SD)	5.5 (±4.9)	5.6 (±5.7)	5.2 (±4.7)	6.2 (±4.8)	0.631	0.654
Discharge NIHSS, mean (±SD)	4.6 (±3.7)	4.8 (±5.5)	4.6 (±4.9)	5.5 (±4.4)	0.685	0.521
**TOAST classification**, ***n*** **(%)^b^**					0.012^*^	0.847
Large artery atherosclerosis	6 (30.0)	31 (41.9)	39 (27.1)	20 (32.3)		
Small vessel occlusion	6 (30.0)	26 (35.1)	76 (52.8)	30 (48.4)		

*CMB, cerebral microbleeds; NIHSS, National Institutes of Health Stroke Scale; SD, standard deviation; TIA, transient ischemic attack; WML, white matter lesions*.

a *Analysis of variance*;

b *Chi-squared test*.

* *p < 0.05*;

** *p < 0.001*.

**Table 3:** Relationship between deep white matter lesions and characteristics of stroke patients with aspirin treatment failure.

**Deep WML**	**Absent**	**Mild**	**Moderate**	**Severe**	***p*-value**	***p* for trend**
*N*	30 (10.0%)	126 (42.0%)	90 (30.0%)	54 (18.0%)		
Age, year, mean (±SD)^a^	58.7 (±13.8)	68.5 (±11.8)	72.1 (±9.6)	73.7 (±10.2)	<0.001^**^	<0.001^**^
Sex, male, *n* (%)^b^	22 (73.3)	92 (73.0)	64 (71.1)	32 (59.3)	0.293	0.104
CMB, mean (±SD)^a^	0.4 (±0.6)	2.3 (±6.1)	3.8 (±7.7)	9.0 (±15.6)	<0.001^**^	<0.001^**^
**Medical history^b^**						
Hypertension, yes, *n* (%)	23 (76.7)	110 (87.3)	80 (88.9)	49 (90.7)	0.278	0.110
Diabetes, yes, *n* (%)	16 (53.3)	70 (55.6)	41 (45.6)	29 (53.7)	0.528	0.575
Hyperlipidemia, yes, *n* (%)	19 (63.3)	88 (69.8)	58 (64.4)	33 (61.1)	0.660	0.426
Stroke/TIA, yes, *n* (%)	22 (73.3)	83 (65.9)	62 (68.9)	45 (83.3)	0.121	0.112
Coronary artery disease, yes, *n* (%)	2 (6.7)	20 (15.9)	21 (23.3)	6 (11.1)	0.095	0.536
Essen stroke risk score, mean (±SD)^a^	4.4 (±1.3)	5.0 (±1.4)	5.2 (±1.1)	5.6 (±1.4)	0.001^*^	<0.001^**^
**Index stroke^a^**						
Initial NIHSS, mean (±SD)	4.8 (±4.3)	5.5 (±5.1)	5.1 (±4.8)	6.7 (±5.5)	0.209	0.127
Discharge NIHSS, mean (±SD)	4.4 (±4.4)	4.6 (±4.8)	4.5 (±4.9)	6.1 (±5.1)	0.190	0.127
**TOAST classification**, ***n*** **(%)^b^**					0.089	0.848
Large artery atherosclerosis	10 (33.3)	43 (34.1)	25 (27.8)	18 (33.3)		
Small vessel occlusion	9 (30.0)	57 (45.2)	48 (53.3)	24 (44.4)		

*CMB, cerebral microbleeds; NIHSS, National Institutes of Health Stroke Scale; SD, standard deviation; TIA, transient ischemic attack; WML, white matter lesions*.

a *Analysis of variance*;

b *Chi-squared test*.

* *p < 0.05*;

** *p < 0.001*.

### CMBs

In total, 236 patients were evaluated for CMBs. CMBs were identified in 124 (52.5%) patients; the mean number of CMBs was 3.8 ± 9.2. Among patients with CMBs, 41 (33.1%) patients had a single lesion, 39 (31.5%) had 2–4 CMBs, and 44 (35.5%) patients had ≥5 CMBs. The comparisons of demographic characteristics and detailed information for the index stroke between the dichotomous and burdens of CMBs are presented in Table 4.

**Table 4:** Relationship between cerebral microbleeds and characteristics of stroke patients with aspirin treatment failure.

	**Cerebral microbleeds (*****N*** **=** **236)**							
	**Dichotomous**			**Burden**					
	**No CMB**	**CMB**	***p*-value**	**0**	**1–4**	**≥5**	***p*-value**	***p* for trend**
*N*	112 (47.5%)	124 (52.5%)		112	80	44		
Age, year, mean (±SD)^a^	68.6 (±13.3)	69.9 (±11.0)	0.419	68.6 (±13.3)	69.4 (±12.0)	70.8 (±8.9)	0.585	0.302
Sex, male, *n* (%)^b^	81 (72.3)	79 (63.7)	0.157	81 (72.3)	51 (63.7)	28 (63.6)	0.368	0.207
Moderate-to-severe PVWML, *n* (%)^b^	64 (57.1)	99 (79.8)	<0.001^**^	64 (57.1)	62 (77.5)	37 (84.1)	0.001^*^	<0.001^**^
Moderate-to-severe DWML, *n* (%)^b^	41 (36.6)	78 (62.9)	<0.001^**^	41 (36.6)	45 (56.3)	33 (75.0)	<0.001^**^	<0.001^**^
**Medical history^b^**								
Hypertension, yes, *n* (%)	95 (84.8)	107 (86.3)	0.748	95 (84.8)	69 (86.3)	38 (86.4)	0.950	0.770
Diabetes, yes, *n* (%)	59 (52.7)	62 (50.0)	0.681	59 (52.7)	44 (55.0)	18 (40.9)	0.297	0.294
Hyperlipidemia, yes, *n* (%)	75 (67.0)	80 (64.5)	0.692	75 (67.0)	53 (66.3)	27 (61.4)	0.796	0.548
Stroke/TIA, yes, *n* (%)	78 (69.6)	88 (71.0)	0.824	78 (69.6)	52 (65.0)	36 (81.8)	0.142	0.275
Coronary artery disease, yes, *n* (%)	21 (18.8)	18 (14.5)	0.382	21 (18.8)	14 (17.5)	4 (9.1)	0.330	0.185
Essen stroke risk score, mean (±SD)^a^	5.2 (±1.4)	5.1 (±1.2)	0.517	5.2 (±1.4)	5.1 (±1.1)	5.0 (±1.4)	0.760	0.474
**Index stroke^b^**								
Initial NIHSS, mean (±SD)	5.9 (±5.0)	5.5 (±5.3)	0.540	5.9 (±5.0)	5.0 (±4.7)	6.2 (±6.4)	0.386	0.695
Discharge NIHSS, mean (±SD)	5.1 (±4.8)	4.7 (±5.0)	0.536	5.1 (±4.8)	4.5 (±4.6)	5.2 (±5.8)	0.601	0.928
**TOAST classification**, ***n*** **(%)^b^**			0.011^*^				0.101	0.118
Large artery atherosclerosis	46 (41.1)	28 (22.6)		46 (41.1)	18 (22.5)	10 (22.7)		
Small vessel occlusion	38 (33.9)	64 (51.6)		38 (33.9)	41 (51.2)	23 (52.3)		

*CMB, cerebral microbleeds; DWML, deep white matter lesions; NIHSS, National Institutes of Health Stroke Scale; PVWML, periventricular white matter lesions; SD, standard deviation; TIA, transient ischemic attack*.

a *Independent student t test and analysis of variance*.

b *Chi-squared test*.

* *p < 0.05*;

** *p < 0.001*.

For associations between CMBs and other parameters, no significant differences in age, sex, vascular risk factors, and ESRS scores were observed between patients with and without CMBs. The presence of CMBs was associated with moderate-to-severe PVWMLs (*p* < 0.001) and DWMLs (*p* < 0.001). There were increasing prevalence of moderate-to-severe PVWMLs and DWMLs along with increasing CMBs burden (*p*_*trend*_ < 0.001 for both). No association was observed between the presence and burden of CMBs and the severity of the index stroke, indicated by initial and discharge NIHSS scores. The distribution of the TOAST stroke subtype differed significantly between patients with and without CMBs (*p* = 0.011). A high prevalence of stroke due to small vessel occlusion was observed in patients with CMBs.

### Index Stroke Outcomes

For the outcome of the index stroke, 27 (9.0%) patients experienced a neurological deterioration, and 158 (52.7%) patients had a favorable functional outcome. The relationships between neurological deterioration, favorable functional outcomes, and SVD severity are presented in Table 5. Neurological deterioration was not associated with age, sex, presence of WMLs and CMBs, and TOAST stroke subtype. Patients with favorable outcomes were significantly younger (*p* < 0.001), and a larger proportion of patients was male (*p* = 0.009) and with low cardiovascular comorbidities (*p* < 0.001), compared to patients with unfavorable outcomes. Furthermore, PVWML and DWML severities were associated with functional outcomes. Patients with severe PVWMLs and DWMLs had less favorable outcomes compared with those with no to moderate PVWMLs and DWMLs (*p* = 0.001 for both). The presence and number of CMBs were not associated with functional outcomes.

**Table 5:** Risk factors and predictors for neurological deterioration and favorable outcome in stroke patients with aspirin treatment failure.

	**Neurological deterioration**		***p*-value**	**Favorable outcome (mRS 0–2)**		***p*-value**
	**Absent**	**Present**		**Absent**	**Present**	
*N*	273 (91.0%)	27 (9.0%)		142 (47.3%)	158 (52.7%)	
Age, year, mean (±SD)^a^	69.3 (±11.8)	71.9 (±12.1)	0.273	73.0 (±11.1)	66.4 (±11.7)	<0.001^**^
Sex, male, *n* (%)^b^	195 (71.4)	15 (55.6)	0.086	89 (62.7)	121 (76.6)	0.009^*^
Essen stroke risk score, mean (±SD)^a^	5.1 (±1.3)	5.4 (±1.2)	0.288	5.4 (±1.3)	4.8 (±1.3)	<0.001^**^
Severe PVWML, *n* (%)^b^	55 (20.1)	7 (25.9)	0.479	41 (28.9)	21 (13.3)	0.001^*^
Severe DWML, *n* (%)^b^	48 (17.6)	6 (22.2)	0.549	37 (26.1)	17 (10.8)	0.001^*^
Presence of CMB, yes, *n* (%)^b^	113 (52.1)	11 (57.9)	0.626	56 (49.1)	68 (55.7)	0.309
Number of CMB, mean (±SD)^a^	4.0 (±9.5)	2.3 (±4.2)	0.455	3.4 (±7.0)	4.2 (±10.9)	0.516
CMB ≥ 5, yes, *n* (%)^b^	41 (18.9)	3 (15.8)	0.739	22 (19.3)	22 (18.0)	0.803
TOAST classification, *n* (%)^b^			0.598			0.584
Large artery atherosclerosis	86 (31.5)	10 (37.0)		47 (33.1)	49 (31.0)	
Small vessel occlusion	127 (46.5)	11 (40.7)		62 (43.7)	76 (48.1)	

*CMB, cerebral microbleeds; DWML, deep white matter lesions; mRS, modified Rankin scale; PVWML, periventricular white matter lesions; SD, standard deviation*.

a *Independent student t-test*.

b *Chi-squared test*.

* *p < 0.05*;

** *p < 0.001*.

After logistic regression was applied, the severe PVWMLs and DWMLs were associated with non-significant risks of increased neurological deterioration. Severe WMLs predicted significantly less favorable outcomes independently, compared with those with no to moderate PVWMLs and DWMLs [odds ratio (OR), 0.47; 95% confidence interval (CI), 0.25–0.87 for severe PVWMLs; OR, 0.40; 95% CI, 0.21–0.79 for severe DWMLs]. The burden of CMBs was not associated with a neurological deterioration and functional outcome (Table 6).

**Table 6:** A multivariable logistic regression analysis to estimate the odds ratio (OR) of burdens of white matter lesions and cerebral microbleeds for neurological deterioration and favorable outcome in stroke patients with aspirin treatment failure.

	**Neurological deterioration**		**Favorable outcome (mRS 0–2)**	
	**OR (95% CI)**	***p*-value^a^**	**OR (95% CI)**	***p*-value^a^**
**PVWML**				
Fazekas 0–2 (*N* = 238)	1 (Reference)		1 (Reference)	
Fazekas 3 (*N* = 62)	1.20 (0.46–3.10)	0.709	0.47 (0.25–0.87)	0.016^*^
**DWML**				
Fazekas 0–2 (*N* = 246)	1 (Reference)		1 (Reference)	
Fazekas 3 (*N* = 54)	1.08 (0.40–2.91)	0.885	0.40 (0.21–0.79)	0.008^*^
**CMB**				
0 (*N* = 112)	1 (Reference)		1 (Reference)	
1–4 (*N* = 80)	1.37 (0.46–4.02)	0.572	1.63 (0.86–3.09)	0.135
≥5 (*N* = 44)	0.91 (0.22–3.81)	0.896	1.23 (0.57–2.64)	0.592

*CI, confidence interval; CMB, cerebral microbleeds; DWML, deep white matter lesions; mRS, modified Rankin scale; PVWML, periventricular white matter lesions*.

a *Multivariable logistic regression analysis, adjusted for age, sex, Essen stroke risk score, and TOAST classification of the index stroke*.

* *p < 0.05*.

## Discussion

The present study reported the prevalence and clinical characteristics of SVD in ATF. The prevalence of PVWMLs, DWMLs and CMBs were 93.3, 90.0, and 52.5%, respectively. Age and cardiovascular comorbidities, particularly hypertension, were major risks for both PVWMLs and DWMLs in ATF. The severity of PVWMLs and DWMLs increased with age. Small vessel occlusion contributed to the most of ischemic stroke with ATF, compared with other TOAST classifications. Both PVWMLs and DWMLs were associated with functional outcomes after stroke. Furthermore, the extent of WMLs was associated with the CMB burden. However, CMBs were not significantly associated with stroke severity, neurological deterioration, or functional outcomes.

ATF is associated with increased cardiovascular event and mortality risks (23). Therefore, patients with ATF require a comprehensive investigation to identify the contributing factors. Different categories of ATF can be distinguished in stroke patients. ATF may exceed functional resistance to aspirin, and could be associated with multiple factors, including advanced age, non-compliance, pharmacodynamic interaction, comorbidities, and undetected cardioembolic sources or atherosclerotic disease (13, 23, 24). In addition, ischemic stroke with ATF could be associated with prior ischemic stroke, particularly lacunar stroke (10, 12, 25). However, to the best of our knowledge, the burden of SVD in stroke patients with ATF has not been well-established.

The prevalence of moderate-to-severe PVWMLs and DWMLs in Asian stroke patients are estimated at 40–53%, and 29–41%, respectively (26, 27). CMBs are present in 20–40% of stroke patients (28). The cohort of stroke with ATF in this study has numerically higher prevalence of WMLs and CMBs than general stroke patients. In addition, a positive relationship between WMLs severity and CMBs burden was demonstrated. The presence and burden of WMLs and CMBs indicate endothelial dysfunction (14), which contribute to platelet activation (29), and then decrease platelet response to aspirin. In addition, the severity of WMLs was positively correlated with cardiovascular comorbidities, which could contribute to aspirin resistance via cyclooxygenase (COX)-1-independent mechanisms (30). The small vessel occlusions of the TOAST classification contributed to 46.0% of stroke after ATF in this study, a higher incidence compared to that of other Asian countries (31–33). Therefore, the high SVD burden could be associated with ATF in stroke patients.

The presence of severe PVWMLs and DWMLs was independently associated with an unfavorable short-term functional outcome in stroke patients with ATF, which was in line with reports regarding outcomes of general stroke patients with WMLs (34, 35). Recovery from stroke involves widespread neural network to compensate for the damaged neural connections (36). SVD are associated with impairment of neural connectivity, which supports the finding that WMLs could contribute to poor functional outcome in stroke patients with ATF.

The optimal antiplatelet regimen for patients with stroke and ATF remains undetermined. Moreover, the safety should be considered when determining antiplatelet regimens in patients with ATF. A meta-analysis suggested that adding to or replacing aspirin with another antiplatelet agent is associated with fewer recurrent stroke and cardiovascular events than maintaining aspirin monotherapy (37). Clopidogrel is the most used alternative antiplatelet agent in ATF. However, the therapeutic efficacy was heterogeneous. Two cohort studies in Asia indicated that dual antiplatelet therapy (DAPT; clopidogrel plus aspirin) or clopidogrel monotherapy was associated with reduced recurrent vascular events in patients with ATF than was aspirin monotherapy (38, 39). However, the Secondary Prevention of Small Subcortical Strokes Trial (SPS3) cohort reported that the addition of clopidogrel to aspirin therapy did not reduce vascular events compared with aspirin monotherapy in patients with a lacunar stroke with ATF (10).

Ticagrelor, a direct-acting antiplatelet agent that reversibly binds and inhibits the P2Y12 receptor on platelets, has been investigated for secondary vascular events prevention after acute ischemic stroke. In ischemic stroke with ATF, treatment with ticagrelor could reduce the risk of recurrent ischemic stroke, but increase combination of major or minor bleedings (40). However, combination of ticagrelor and aspirin does not decrease the incidence of composite of stroke or death and disabling stroke compared with aspirin after ischemic stroke with ATF (41, 42).

Cilostazol was the second most commonly used antiplatelet agent after ATF to add to or replace aspirin in an Asian cohort (39). The cilostazol monotherapy reduced recurrent, ischemic and hemorrhagic stroke compared with aspirin, and cilostazol combination with aspirin or clopidogrel did not increase hemorrhagic stroke prevalence in patients with non-cardioembolic stroke (43).

The burden of SVD has a predictive value for ICH after TIA or ischemic stroke (4), particularly among those with multiple CMBs receiving treatment with antiplatelet agents (44). Therefore, the optimal antiplatelet strategy after ATF warrants further consideration, given the SVD burden. Cilostazol reduced hemorrhagic stroke prevalence in patients with multiple CMBs and reduced total stroke in patients with mild-to-moderate WMLs compared with aspirin (45). However, evidence on the safety of antiplatelet regimens in patients combined with ATF and SVD is scarce.

The novelty of this study is that the clinical association between SVD and ATF was well-presented. The association between WMLs and poor functional outcome in stroke patients with ATF was shown to be significant. However, there are several limitations to our study. First, this study adapted a cross-sectional and observational design, and there is no control group in this registry. Therefore, it limited the interpretation of causal association of SVD on ATF. In addition, we could not determine the long-term effect of SVD on stroke recurrence and cognition after adjustment of antiplatelet regimens. Second, all patients in this study were Taiwanese, limiting the generalizability to other populations. Third, the registry enrolled stroke patients taking aspirin regularly 7 days before the index stroke. However, the precise duration of aspirin treatment before the index stroke was not recorded.

In conclusion, WMLs severity is highly correlated with age, cardiovascular comorbidities and CMBs burden. PVWMLs and DWMLs are significantly associated with functional outcomes in stroke patients with ATF. Further antiplatelet strategy after ATF considering the burden of SVD should be warranted.

## Data Availability Statement

The raw data supporting the conclusions of this article will be made available by the authors, without undue reservation.

## Ethics Statement

The studies involving human participants were reviewed and approved by Institutional Review Board of all centers in this study. The patients/participants provided their written informed consent to participate in this study.

## Author Contributions

H-HH and A-CC: study concept, design, and supervision. P-SC, P-SS, C-HLiu, Y-FS, R-CT, C-HLie, HP, S-CH, Y-TT, T-SC, and S-LW: acquisition and integrity of data. P-SC, P-SS, C-HLiu, and Y-FS: analysis and interpretation of data, statistical analysis and drafting the article. All authors contributed to the critical revision of the article for important intellectual content, agreed to the final version of the manuscript.

## Conflict of Interest

The authors declare that the research was conducted in the absence of any commercial or financial relationships that could be construed as a potential conflict of interest.

## References

[1] Katan M Luft A. Global burden of stroke. Semin Neurol. (2018) 38:208–11. 10.1055/s-0038-1649503 29791947

[2] Donkor ES. Stroke in the 21(st) century: a snapshot of the burden, epidemiology, and quality of life. Stroke Res Treat. (2018) 2018:3238165. 10.1155/2018/3238165 30598741 PMC6288566

[3] Pantoni L. Cerebral small vessel disease: from pathogenesis and clinical characteristics to therapeutic challenges. Lancet Neurol. (2010) 9:689–701. 10.1016/S1474-4422(10)70104-6 20610345

[4] Lau KK Li L Schulz U Simoni M Chan KH Ho SL. Total small vessel disease score and risk of recurrent stroke: Validation in 2 large cohorts. Neurology. (2017) 88:2260–7. 10.1212/WNL.0000000000004042 28515266 PMC5567324

[5] Kernan WN Ovbiagele B Black HR Bravata DM Chimowitz MI Ezekowitz MD. Guidelines for the prevention of stroke in patients with stroke and transient ischemic attack: a guideline for healthcare professionals from the American heart association/American stroke association. Stroke. (2014) 45:2160–236. 10.1161/STR.0000000000000024 24788967

[6] Guzik A Bushnell C. Stroke epidemiology and risk factor management. Continuum. (2017) 23:15–39. 10.1212/CON.0000000000000416 28157742

[7] Baigent C Blackwell L Collins R Emberson J Godwin J Peto R. Aspirin in the primary and secondary prevention of vascular disease: collaborative meta-analysis of individual participant data from randomised trials. Lancet. (2009) 373:1849–60. 10.1016/S0140-6736(09)60503-1 19482214 PMC2715005

[8] National Institute of Neurological Disorders and Stroke rt-PA Stroke Study Group. Tissue plasminogen activator for acute ischemic stroke. N Engl J Med. (1995) 333:1581–7. 10.1056/NEJM199512143332401 7477192

[9] The Publications Committee for the Trial of ORG 10172 in Acute Stroke Treatment (TOAST) Investigators. Low molecular weight heparinoid, ORG 10172 (danaparoid), and outcome after acute ischemic stroke: a randomized controlled trial. JAMA. (1998) 279:1265–72. 10.1001/jama.279.16.1265 9565006

[10] Cote R Zhang Y Hart RG McClure LA Anderson DC Talbert RL. ASA failure: does the combination ASA/clopidogrel confer better long-term vascular protection? Neurology. (2014) 82:382–9. 10.1212/WNL.0000000000000076 24384643 PMC3917683

[11] Qureshi AI Kirmani JF Safdar A Ahmed S Sayed MA Pande RU. High prevalence of previous antiplatelet drug use in patients with new or recurrent ischemic stroke: buffalo metropolitan area and Erie county stroke study. Pharmacotherapy. (2006) 26:493–8. 10.1592/phco.26.4.493 16553507

[12] Pujol Lereis VA Ameriso S Povedano GP Ameriso SF. Ischemic stroke in patients receiving aspirin. J Stroke Cerebrovasc Dis. (2012) 21:868–72. 10.1016/j.jstrokecerebrovasdis.2011.05.009 21703876

[13] Gallo A Galliazzo S Grazioli S Guasti L Ageno W Squizzato A. Epidemiology and secondary prevention of ischemic stroke in patients on antiplatelet drug: a retrospective cohort study. J Thromb Thrombolysis. (2019) 48:336–44. 10.1007/s11239-019-01893-y 31177486

[14] Quick S Moss J Rajani RM Williams A. A vessel for change: endothelial dysfunction in cerebral small vessel disease. Trends Neurosci. (2020) 44:289–305. 10.1016/j.tins.2020.11.003 33308877

[15] Petersen M Frey BM Schlemm E Mayer C Hanning U Engelke K. Network localisation of white matter damage in cerebral small vessel disease. Sci Rep. (2020) 10:9210. 10.1038/s41598-020-66013-w 32514044 PMC7280237

[16] CAPRIE Steering Committee. A randomised, blinded, trial of clopidogrel versus aspirin in patients at risk of ischaemic events (CAPRIE). CAPRIE Steering Committee. Lancet. (1996) 348:1329–39. 10.1016/S0140-6736(96)09457-3 8918275

[17] Weimar C Benemann J Michalski D Muller M Luckner K Katsarava Z. Prediction of recurrent stroke and vascular death in patients with transient ischemic attack or nondisabling stroke: a prospective comparison of validated prognostic scores. Stroke. (2010) 41:487–93. 10.1161/STROKEAHA.109.562157 20056932

[18] Fitzek S Leistritz L Witte OW Heuschmann PU Fitzek C. The essen stroke risk score in one-year follow-up acute ischemic stroke patients. Cerebrovasc Dis. (2011) 31:400–7. 10.1159/000323226 21346341

[19] Adams HP Jr Bendixen BH Kappelle LJ Biller J Love BB. Classification of subtype of acute ischemic stroke. Definitions for use in a multicenter clinical trial. TOAST. trial of Org 10172 in acute stroke treatment. Stroke. (1993) 24:35–41. 10.1161/01.STR.24.1.35 7678184

[20] Fazekas F Chawluk JB Alavi A Hurtig HI Zimmerman RA. MR signal abnormalities at 1.5 T in alzheimer's dementia and normal aging. AJR Am J Roentgenol. (1987) 149:351–6. 10.2214/ajr.149.2.351 3496763

[21] Pantoni L Basile AM Pracucci G Asplund K Bogousslavsky J Chabriat H. Impact of age-related cerebral white matter changes on the transition to disability – the LADIS study: rationale, design and methodology. Neuroepidemiology. (2005) 24:51–62. 10.1159/000081050 15459510

[22] Gregoire SM Chaudhary UJ Brown MM Yousry TA Kallis C Jager HR. The microbleed anatomical rating scale (MARS): reliability of a tool to map brain microbleeds. Neurology. (2009) 73:1759–66. 10.1212/WNL.0b013e3181c34a7d 19933977

[23] Maree AO Fitzgerald DJ. Variable platelet response to aspirin and clopidogrel in atherothrombotic disease. Circulation. (2007) 115:2196–207. 10.1161/CIRCULATIONAHA.106.675991 17452618

[24] Hankey GJ Eikelboom JW. Aspirin resistance. Lancet. (2006) 367:606–17. 10.1016/S0140-6736(06)68040-9 16488805

[25] Englyst NA Horsfield G Kwan J Byrne CD. Aspirin resistance is more common in lacunar strokes than embolic strokes and is related to stroke severity. J Cereb Blood Flow Metab. (2008) 28:1196–203. 10.1038/jcbfm.2008.9 18319729

[26] Chou PS Chen CH Wu MN Lin YH Lai CL Lin RT. Determinants of cerebral white matter changes in patients with stroke. Intern Med J. (2015) 45:390–5. 10.1111/imj.12704 25644475

[27] Xu YY Zong LX Zhang CQ Pan YS Jing J Meng X. The association of white matter hyperintensities with stroke outcomes and antiplatelet therapy in minor stroke patients. Ann Transl Med. (2020) 8:331. 10.21037/atm.2020.02.137 32355775 PMC7186621

[28] Kim BJ Lee SH. Prognostic impact of cerebral small vessel disease on stroke outcome. J Stroke. (2015) 17:101–110. 10.5853/jos.2015.17.2.101 26060797 PMC4460329

[29] Hamilos M Petousis S Parthenakis F. Interaction between platelets and endothelium: from pathophysiology to new therapeutic options. Cardiovasc Diagn Ther. (2018) 8:568–80. 10.21037/cdt.2018.07.01 30498682 PMC6232347

[30] Feher G Feher A Pusch G Koltai K Tibold A Gasztonyi B. Clinical importance of aspirin and clopidogrel resistance. World J Cardiol. (2010) 2:171–86. 10.4330/wjc.v2.i7.171 21160749 PMC2998916

[31] Jung KH Lee SH Kim BJ Yu KH Hong KS Lee BC. Secular trends in ischemic stroke characteristics in a rapidly developed country: results from the Korean stroke registry study (secular trends in Korean stroke). Circ Cardiovasc Qual Outcomes. (2012) 5:327–34. 10.1161/CIRCOUTCOMES.111.963736 22474244

[32] Hsieh FI Chiou HY. Stroke: morbidity, risk factors, and care in taiwan. J Stroke. (2014) 16:59–64. 10.5853/jos.2014.16.2.59 24949310 PMC4060269

[33] Tian D Yang Q Dong Q Li N Yan B Fan D. Trends in stroke subtypes and vascular risk factors in a stroke center in China over 10 years. Sci Rep. (2018) 8:5037. 10.1038/s41598-018-23356-9 29567985 PMC5864718

[34] Liou LM Chen CF Guo YC Cheng HL Lee HL Hsu JS. Cerebral white matter hyperintensities predict functional stroke outcome. Cerebrovasc Dis. (2010) 29:22–7. 10.1159/000255970 19893308

[35] Griessenauer CJ McPherson D Berger A Cuiper P Sofoluke N Adams MD. Effects of white matter hyperintensities on 90-day functional outcome after large vessel and non-large vessel stroke. Cerebrovasc Dis. (2020) 49:419–26. 10.1159/000509071 32694259 PMC7501232

[36] Di Filippo M Tozzi A Costa C Belcastro V Tantucci M Picconi B. Plasticity and repair in the post-ischemic brain. Neuropharmacology. (2008) 55:353–62. 10.1016/j.neuropharm.2008.01.012 18359495

[37] Lee M Saver JL Hong KS Rao NM Wu YL Ovbiagele B. Antiplatelet regimen for patients with breakthrough strokes while on aspirin: a systematic review and meta-analysis. Stroke. (2017) 48:2610–3. 10.1161/STROKEAHA.117.017895 28701574

[38] Lee M Wu YL Saver JL Lee HC Lee JD Chang KC. Is clopidogrel better than aspirin following breakthrough strokes while on aspirin? A retrospective cohort study. BMJ Open. (2014) 4:e006672. 10.1136/bmjopen-2014-006672 25468508 PMC4256539

[39] Kim JT Park MS Choi KH Cho KH Kim BJ Han MK. Different antiplatelet strategies in patients with new ischemic stroke while taking aspirin. Stroke. (2016) 47:128–34. 10.1161/STROKEAHA.115.011595 26604247

[40] Wong KSL Amarenco P Albers GW Denison H Easton JD Evans SR. Efficacy and safety of ticagrelor in relation to aspirin use within the week before randomization in the SOCRATES trial. Stroke. (2018) 49:1678–85. 10.1161/STROKEAHA.118.020553 29915123 PMC6019568

[41] Johnston SC Amarenco P Denison H Evans SR Himmelmann A James S. Ticagrelor and aspirin or aspirin alone in acute ischemic stroke or TIA. N Engl J Med. (2020) 383:207–17. 10.1056/NEJMoa1916870 32668111

[42] Amarenco P Denison H Evans SR Himmelmann A James S Knutsson M. Ticagrelor added to aspirin in acute ischemic stroke or transient ischemic attack in prevention of disabling stroke: a randomized clinical trial. JAMA Neurol. (2020) 78:1–9. 10.1001/jamaneurol.2020.4396 33159526 PMC7648910

[43] Kim SM Jung JM Kim BJ Lee JS Kwon SU. Cilostazol mono and combination treatments in ischemic stroke: an updated systematic review and meta-analysis. Stroke. (2019) 50:3503–11. 10.1161/STROKEAHA.119.026655 31607242

[44] Lau KK Lovelock CE Li L Simoni M Gutnikov S Kuker W. Antiplatelet treatment after transient ischemic attack and ischemic stroke in patients with cerebral microbleeds in 2 large cohorts and an updated systematic review. Stroke. (2018) 49:1434–42. 10.1161/STROKEAHA.117.020104 29748422 PMC5976229

[45] Kim BJ Kwon SU Park JH Kim YJ Hong KS Wong LKS. Cilostazol versus aspirin in ischemic stroke patients with high-risk cerebral hemorrhage: subgroup analysis of the PICASSO trial. Stroke. (2020) 51:931–7. 10.1161/STROKEAHA.119.023855 31856691

